# Prognostic value of tumour microenvironment‐related genes by TCGA database in rectal cancer

**DOI:** 10.1111/jcmm.16547

**Published:** 2021-05-05

**Authors:** Chao Li, Tao Liu, Yi Liu, Jiantao Zhang, Didi Zuo

**Affiliations:** ^1^ Department of Colorectal and Anal Surgery The First Hospital of Jilin University Changchun China; ^2^ Department of Endocrinology and Metabolism The First Hospital of Jilin University Changchun China

**Keywords:** ESTIMATE algorithm, overall survival, prognosis, rectal cancer, tumour microenvironment

## Abstract

Rectal cancer is a common malignant tumour and the progression is highly affected by the tumour microenvironment (TME). This study intended to assess the relationship between TME and prognosis, and explore prognostic genes of rectal cancer. The gene expression profile of rectal cancer was obtained from TCGA and immune/stromal scores were calculated by Estimation of Stromal and Immune cells in Malignant Tumors using Expression data (ESTIMATE) algorithm. The correlation between immune/stromal scores and survival time as well as clinical characteristics were evaluated. Differentially expressed genes (DEGs) were identified according to the stromal/immune scores, and the functional enrichment analyses were conducted to explore functions and pathways of DEGs. The survival analyses were conducted to clarify the DEGs with prognostic value, and the protein‐protein interaction (PPI) network was performed to explore the interrelation of prognostic DEGs. Finally, we validated prognostic DEGs using data from the Gene Expression Omnibus (GEO) database by PrognoScan, and we verified these genes at the protein levels using the Human Protein Atlas (HPA) databases. We downloaded gene expression profiles of 83 rectal cancer patients from The Cancer Genome Atlas (TCGA) database. The Kaplan‐Meier plot demonstrated that low‐immune score was associated with worse clinical outcome (*P* = .034), metastasis (M1 vs. M0, *P* = .031) and lymphatic invasion (+ vs. ‐, *P* < .001). A total of 540 genes were screened as DEGs with 539 up‐regulated genes and 1 down‐regulated gene. In addition, 60 DEGs were identified associated with overall survival. Functional enrichment analyses and PPI networks showed that the DEGs are mainly participated in immune process, and cytokine‐cytokine receptor interaction. Finally, 19 prognostic genes were verified by GSE17536 and GSE17537 from GEO, and five genes (*ADAM23, ARHGAP20, ICOS, IRF4,* *MMRN1*) were significantly different in tumour tissues compared with normal tissues at the protein level. In summary, our study demonstrated the associations between TME and prognosis as well as clinical characteristics of rectal cancer. Moreover, we explored and verified microenvironment‐related genes, which may be the potential key prognostic genes of rectal cancer. Further clinical samples and functional studies are needed to validate this finding.

## 1 INTRODUCTION

Rectal cancer is a common malignant tumour of gastrointestinal tract which occurs in the lower part of the colon. Rectal cancer and colon cancer are often grouped as ‘colorectal cancer’(CRC), which is the third leading cause of cancer‐related deaths in the world. There were 1.4 million new CRC cases every year, and this figure will rise in the future. It is expected to increase to 2.2 million CRC cases and 1.1 million deaths in ten years,^1^ of which 35% were rectal cancer.^2^ In China, the rate of rectal cancer was 11.45 and 8.28 per 100,000 in men and females, respectively.^3^ Over the past 30 years, effective screening measures and multimodal therapies had depressed the incidence and mortality rate and improved long‐term survival rates. However, there were 80% of CRC patients show recurrence in the first 3 years. Thus, identifying reliable prognostic biomarkers to select rectal cancer patients at high risk for recurrence is important for improving the survival rate.

TME consists of tumour cells, stromal cells, immune cells and extracellular matrix, which influences cancer growth and development significantly. Tumour cells in the TME can invade tissues directly or through blood and lymphatic vessels, and the infiltrated cells can induce the immune response by releasing cytokines, cytokine receptors and other factors, which influenced the progression of tumour.^4^ In recent years, new studies revealed that TME significantly affect the progression of tumours, and have shown a potential predictive value for cancer prognosis,^5^, ^6^, ^7^, ^8^, ^9^, ^10^, ^11^ including CRC.^5^, ^12^

With the rapid development of precision medicine, researchers are increasingly exploring new diagnosis and the treatment targets using statistical algorithms. TCGA provided genomic profiles and clinical information, making it possible to investigate the correlation between genomic features and clinical as well as prognostic characteristics.^13^ ESTIMATE is an algorithm which was raised to evaluate the role of stromal and immune cells in cancer biology. ESTIMATE algorithm is a tool assessing stromal score, immune score and estimate score (that infers tumour purity) in tumour tissues by using gene expression data.

TCGA database and the ESTIMATE algorithm has been widely applied to investigate cancer prognosis prediction. Recent studies showed ESTIMATE has good precision in hepatocellular carcinoma, renal cell carcinoma and glioblastoma,^14^, ^15^, ^16^, ^17^, ^18^ but it has not been applied for rectal cancer. Therefore, we intend to identify TME‐related genes that significantly affect rectal cancer prognosis by ESTIMATE and TCGA database.

## 2 METHODS

### 2.1 Data source

We downloaded gene expression profile and survival information as well as clinical features of rectal cancer patients from TCGA database (https://tcga‐data.nci.nih.gov/tcga). The clinical features include age, gender, race, TNM status, survival status, values of carcinoembryonic antigen (CEA), venous invasion, lymphatic invasion and perineural invasion condition. We downloaded all the data from TCGA, and performed data acquirement and application following TCGA guidelines.

### 2.2 Survival analysis and DEGs identification

To explore the correlation between stromal or immune scores and prognosis of rectal cancer patients, we performed survival analysis with survival time. Immune and stromal scores were calculated by ESTIMATE algorithm, and categorized into high‐ and low‐score group according to the median of immune/stromal scores. We performed the analyses using R package ‘limma’ (version 3.44.1), with the following cut‐off value: log fold change (FC) > 1.0 and false discovery rate (FDR) <0.05. Heatmaps were performed using R package ‘pheatmap’ (version 1.12.0) package. To identify the predictive DEGs in overall survival (OS) of rectal cancer, we constructed Kaplan‐Meier plots.

### 2.3 Functional enrichment analyses

Gene Ontology (GO) and Kyoto Encyclopedia of Genes and Genomes (KEGG) enrichment analysis was performed to reveal the functions and associated pathways of DEGs, with ‘cluster profile’ (version 3.17.0), ‘org. Hs.eg.db’ (version 3.11.1), ‘enrichplot’ (version 1.8.1) and ‘ggplot2’ (version 3.3.0) on R.

### 2.4 PPI network construction

To understand the interactions between prognostic DEGs, we constructed the PPI network by an opensource software platform Search Tool for the Retrieval of Interacting Genes (STRING, https://string‐db.org/). Then the modular analysis was performed by CytoHubba plug‐in in the Cytoscape software (version 3.7.1), and the most significant modules were identified based on the score and node number.

### 2.5 Verification of DEGs using GEO database and clinical tissue samples

To verify the prognostic DEGs from TCGA, we downloaded the gene expression and prognostic data from GSE17536 and GSE17537 data sets using PrognoScan online tool.^19^ To further confirm the reliability of the prognostic DEGs, we detected the antibody‐based protein expression data in normal tissues and tumour tissues from The Human Protein Atlas database (HPA, www.proteinatlas.org.).

### 2.6 Expression of hub genes in cell types

The Single Cell Type Atlas part in HPA showed the expression of protein‐coding genes in single human cell types, and the number of genes detected in cell types. The mRNA and protein levels of hub genes expression in cell types were evaluated using this tool.

### 2.7 Statistical analysis

The Kaplan‐Meier survival analyses was used to illuminate correlations between expression of DEGs and the OS of rectal cancer, and identify prognostic DEGs in overall survival. Univariate analyses were performed between clinical characteristics and stromal/immune scores. All statistical tests were done with R (version 3.6.2). *P* < .05 was regarded as statistically significant.

## 3 RESULTS

### 3.1 Stromal and Immune Scores of the Patients

The gene expression profiles and clinical information of 83 rectal cancer patients were downloaded from TCGA database. Based on the ESTIMATE algorithm, stromal score ranges from −1979.57 to 1,522.96, and immune score ranges from −656.67 to 2,102.23 (Table 1).

**TABLE 1:** Immune scores, stromal scores and clinical characteristics of patients with rectal cancer

Characteristic	No.	Percent (%)	Stromal score range	Immune score range
Age				
>60	52	62.65	‐1979.57 to 1522.96	‐627.18 to 2102.23
≤60	31	37.35	‐1647.38 to 437.50	‐656.67 to 1634.65
Gender				
Male	47	56.63	‐1831.95 to 736.53	‐656.67 to 1634.65
Female	36	43.37	‐1979.57 to 1522.96	‐627.18 to 2102.23
TNM Stage				
I	17	20.48	‐1500.59 to 710.82	‐656.67 to 1333.14
II	25	30.12	‐1440.11 to 353.92	‐477.23 to 1215.29
III	23	27.71	‐1979.57 to 1522.96	‐627.18 to 2102.23
IV	13	15.66	‐1831.95 to 736.53	‐650.76 to 1365.69
Unknown	4	4.82	‐1750.89 to −256.74	‐239.09 to 169.38
T stage				
T1	4	4.82	‐1500.59 to −727.389	‐262.74 to 1308.84
T2	17	20.48	‐1979.57 to 710.82	‐656.67 to 1333.14
T3	56	67.47	‐1831.95 to 1522.96	‐650.76 to 2102.23
T4	5	6.02	‐1167.63 to −242.44	‐627.18 to 1215.29
N stage				
N0	44	53.01	‐1750.89 to 710.82	‐656.67 to 1333.14
N1	25	30.12	‐1979.57 to 736.52	‐627.18 to 1634.65
N2	12	14.46	‐1415.65 to 1522.96	‐650.76 to 2102.23
Nx	1	1.20	‐256.74 to −256.74	121. 05 to 121.42
M stage				
M0	63	75.90	‐1879.57 to 1622.96	‐556.67 to 2202.23
M1	13	15.66	‐1931.95 to 636.53	‐750.76 to 1265.69
Mx	5	6.02	‐1750.89 to 119.47	77.57 to 1215.29
Unknown	1	1.20	‐53.40 to −53.40	1196.84 to 1196.84
Survival status				
Death	72	86.75	‐1750.89 to 1522.96	‐656.67 to 2102.23
Alive	10	12.05	‐1979.57 to 24.80	‐627.18 to 557.27
CEA				
≤5	32	38.55	‐1647.38 to 1522.96	‐267.87 to 2102.23
>5	19	22.89	‐1750.89 to 736.53	‐477.23 to 1634.65
Unknown	31	37.35	‐1979.57 to 450.95	‐656.67 to 1308.84
Race				
White	37	44.58	‐1979.57 to 1522.96	‐627.18 to 2102.23
Black	1	1.20	‐388.15 to −388.15	49.44 to 49.44
Unknown	44	53.01	‐1647.38 to 736.53	‐656.67 to 1396.93
Venous invasion				
YES	20	24.10	‐1562.90 to 1522.96	‐477.23 to 2102.23
No	46	55.42	‐1750.89 to 450.95	‐650.76 to 1634.65
Unknown	16	19.28	‐1979.57 to 736.53	‐656.67 to 1365.69
Lymphatic invasion				
Yes	32	38.55	‐1697.38 to 1472.96	‐700.76 to 2052.23
No	37	44.58	‐1700.89 to 760.82	‐577.18 to 1588.38
Unknown	13	15.66	‐1979.57 to 736.53	‐656.67 to 1365.69
Perineural Invasion				
Yes	8	9.64	‐1750.89 to 710.82	‐232.18 to 1634.65
No	19	22.89	‐1440.11 to 450.95	‐627.18 to 1538.38
Unknown	55	66.27	‐1979.57 to 1522.96	‐656.67 to 2102.23

**FIGURE 1:**
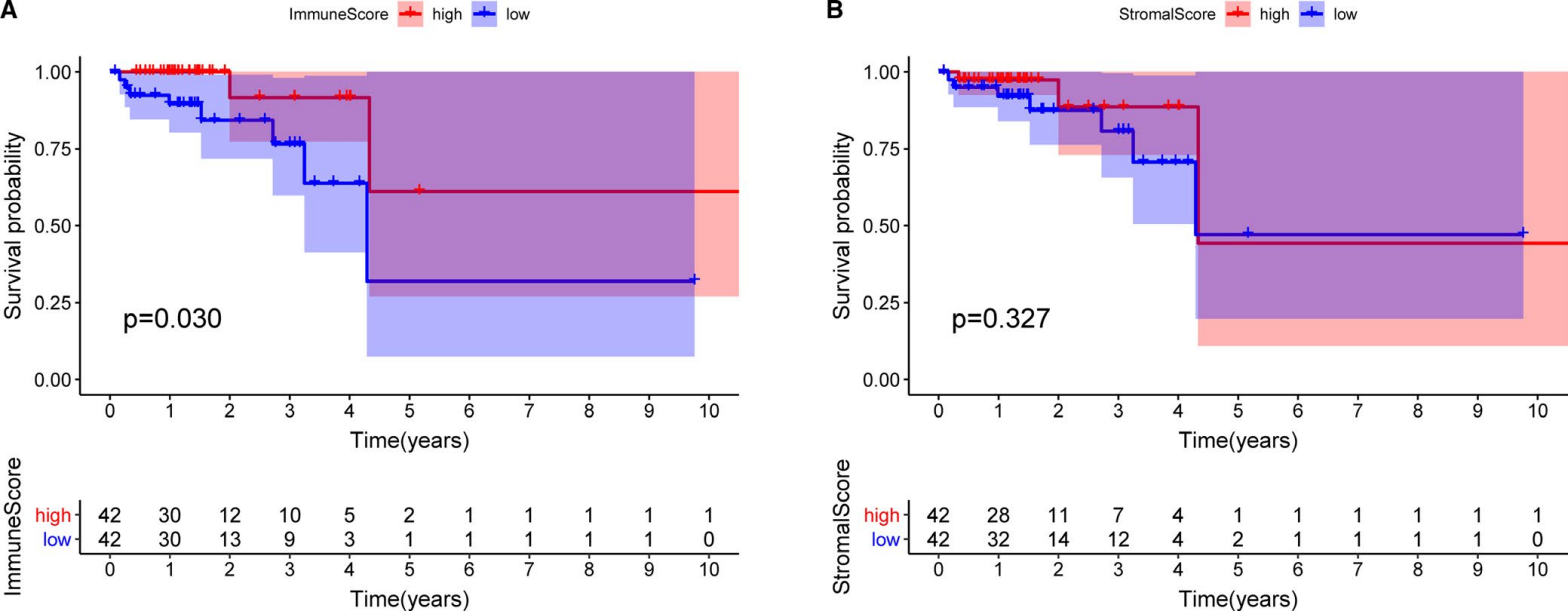
Overall survival curves obtained by the Kaplan‐Meier method describing the correlation between immune scores or stromal scores and prognosis of patients. A, Immune score was significantly associated with overall survival (*P* = .034). B, There was no significantly correlation between stromal score and overall survival (*P* = .316). Horizontal and vertical axes represent survival times and survival rates, respectively. Red and blue curves represent high and low score group, respectively

To explore the association between OS and immune or stromal scores, we classified the 83 rectal cancer patients into high‐ and low‐score groups based on the median of stromal scores (−636.30) and immune scores (268.92). Kaplan‐Meier analysis was performed and the survival curves showed that patients in the high‐immune score group had a better prognosis than those in the low‐immune score group (*P* = .034, Figure 1A). However, there were no statistical differences between high‐stromal score group and low‐stromal score group (*P* = .316, Figure 1B).

**FIGURE 2:**
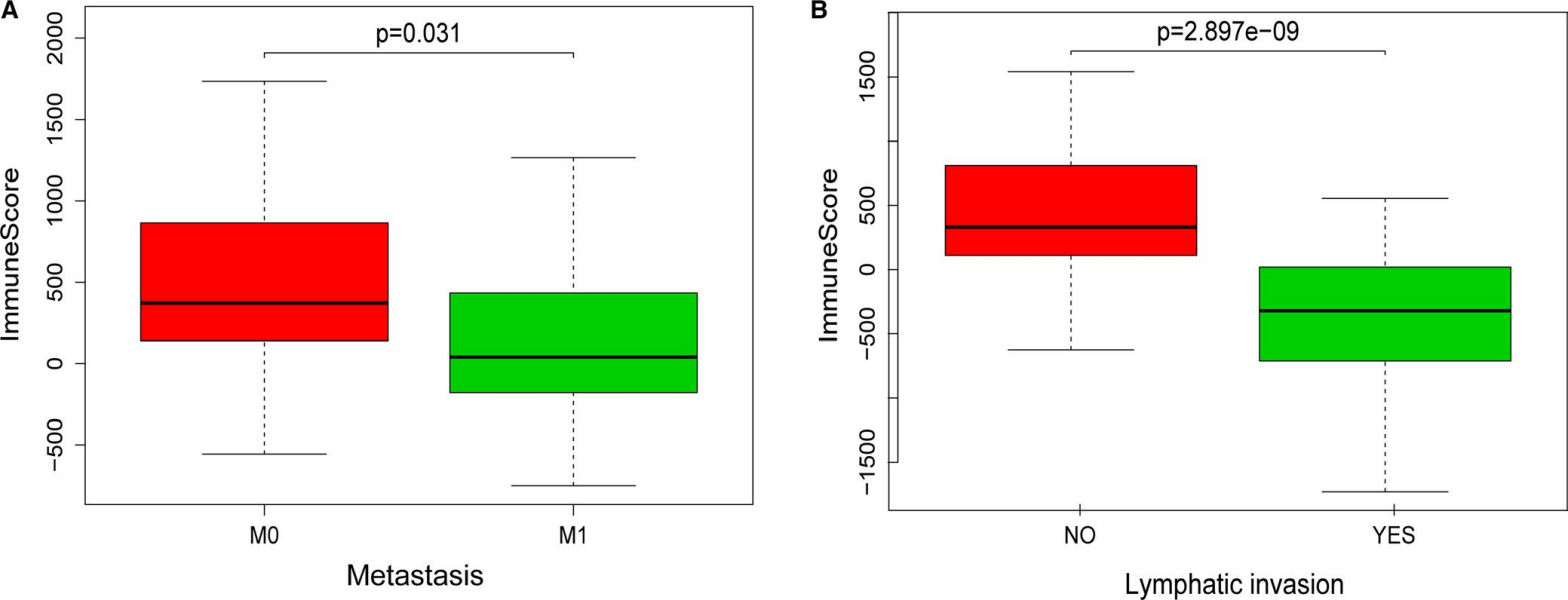
The relationship between immune score and clinical characteristics of patients. A, Low‐immune score was associated with metastasis (M1 vs. M0, *P* = .031). B, Low‐immune score was associated with lymphatic invasion (+ vs. ‐, *P* < .001)

In addition, we analysed the relationship between immune or stromal scores and clinical characteristics. We found that low‐immune score was associated with M1 (vs. M0, *P* = .031, Figure 2A), and lymphatic invasion (+ vs. ‐, *P* < .001, Figure 2B), indicating that lower immune scores indicated the advanced rectal cancer stage. However, there were no evidence to support significant correlation between stromal/ immune scores and T status, N status, CEA value, venous or perineural invasion. (Figure S1, *P* >.05).

### 3.2 Identification of DEGs

**FIGURE 3:**
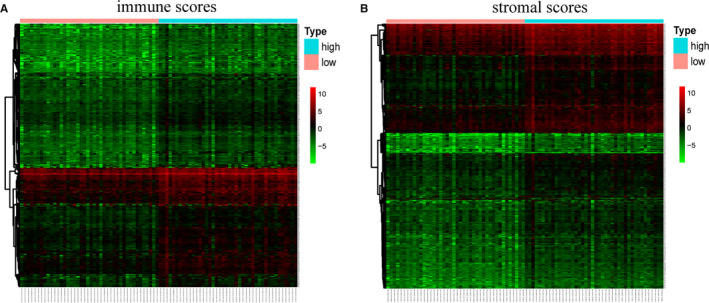
Heatmap of DEGs between high and low groups in immune scores and stromal scores. A, Immune scores (low score in left and high score in right); B, Stromal scores (low score in left and high score in right)

**FIGURE 4:**
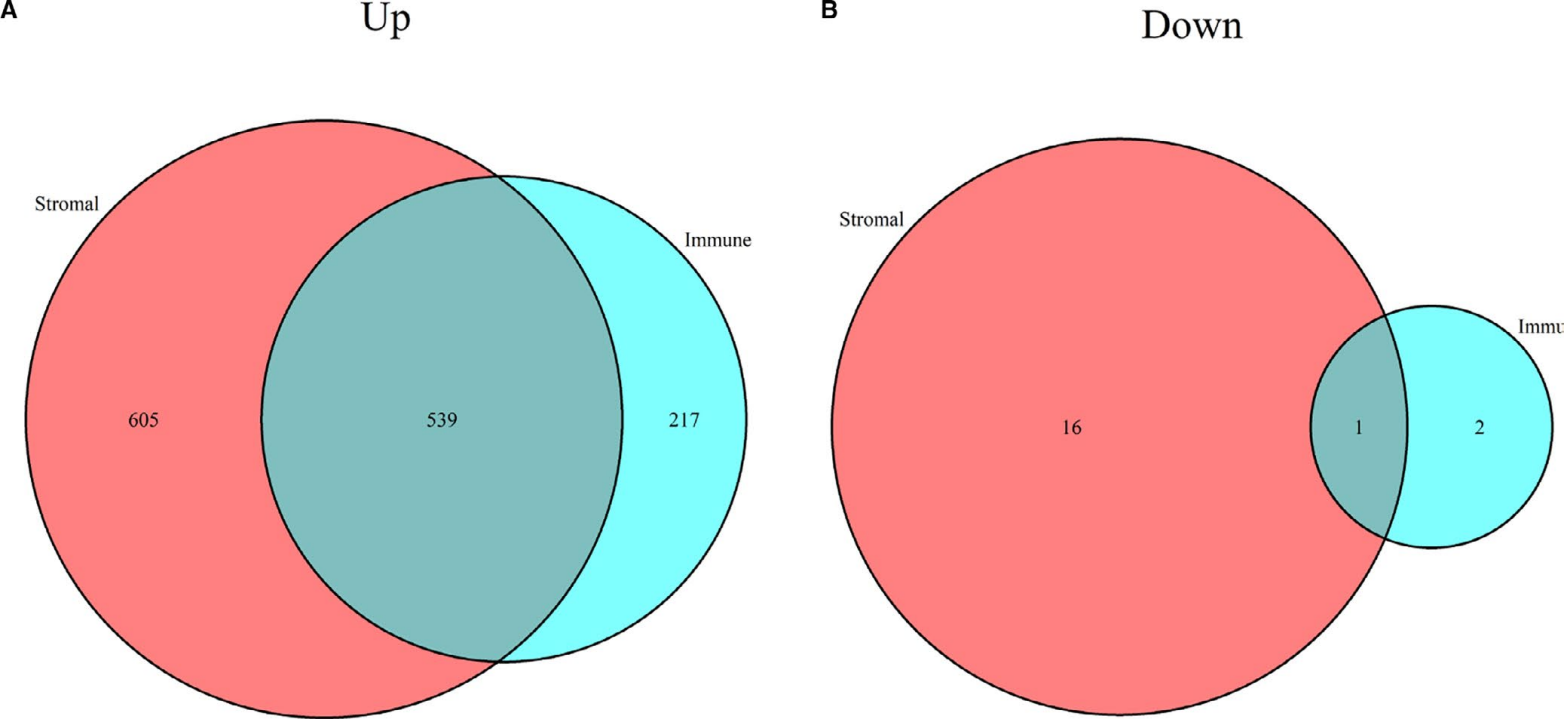
Common up‐regulated or down‐regulated DEGs in immune scores and stromal scores. A, 539 common up‐regulated genes in immune scores and stromal scores; B, 1 common down‐regulated gene in immune scores and stromal scores

To determine the DEGs associated with TME of rectal cancer, we analysed and compared the gene expression profiles of cases in high‐ and low‐immune/stromal score groups. For immune scores, 756 up‐regulated genes and 3 down‐regulated genes were identified (Figure 3A). Similarly, for stromal scores, 1144 up‐regulated genes and 17 down‐regulated genes were identified (Figure 3B). Then, we analysed the shared up‐regulated and down‐regulated genes in immune and stromal score groups, with 539 up‐regulated and 1 down‐regulated gene identified which is shown in Venn diagrams (Figure 4). Totally, there were 540 genes were screened as DEGs.

### 3.3 GO function and KEGG pathway enrichment analyses

**FIGURE 5:**
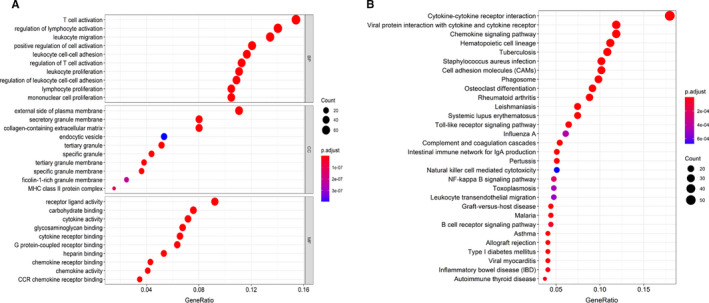
GO functional enrichment analyses and KEGG pathway analyses of DEGs. A, The top 30 significantly enriched GO terms, including biological process (BP), molecular function (MF) and cellular component (CC). B, KEGG pathway analyses of DEGs

The GO function analyses consisted of biological processes (BP), cellular component (CC) and molecular function (MF). For BP, DEGs were mainly enriched in the T cell and lymphocyte activation, leukocyte migration, proliferation and cell‐cell adhesion. For CC, DEGs mainly clustered in the plasma membrane, granule and granule membrane, collagen‐containing extracellular matrix and endocytic vesicle. For MF, DEGs mainly concentrate on receptor ligand activity, receptor binding, cytokine activity and chemokine activity. (Figure 5A). The KEGG analysis indicated that these DEGs were enriched in cytokine‐cytokine receptor interaction and chemokine signalling pathways. (Figure 5B).

### 3.4 Survival analysis of the DEGs

**FIGURE 6:**
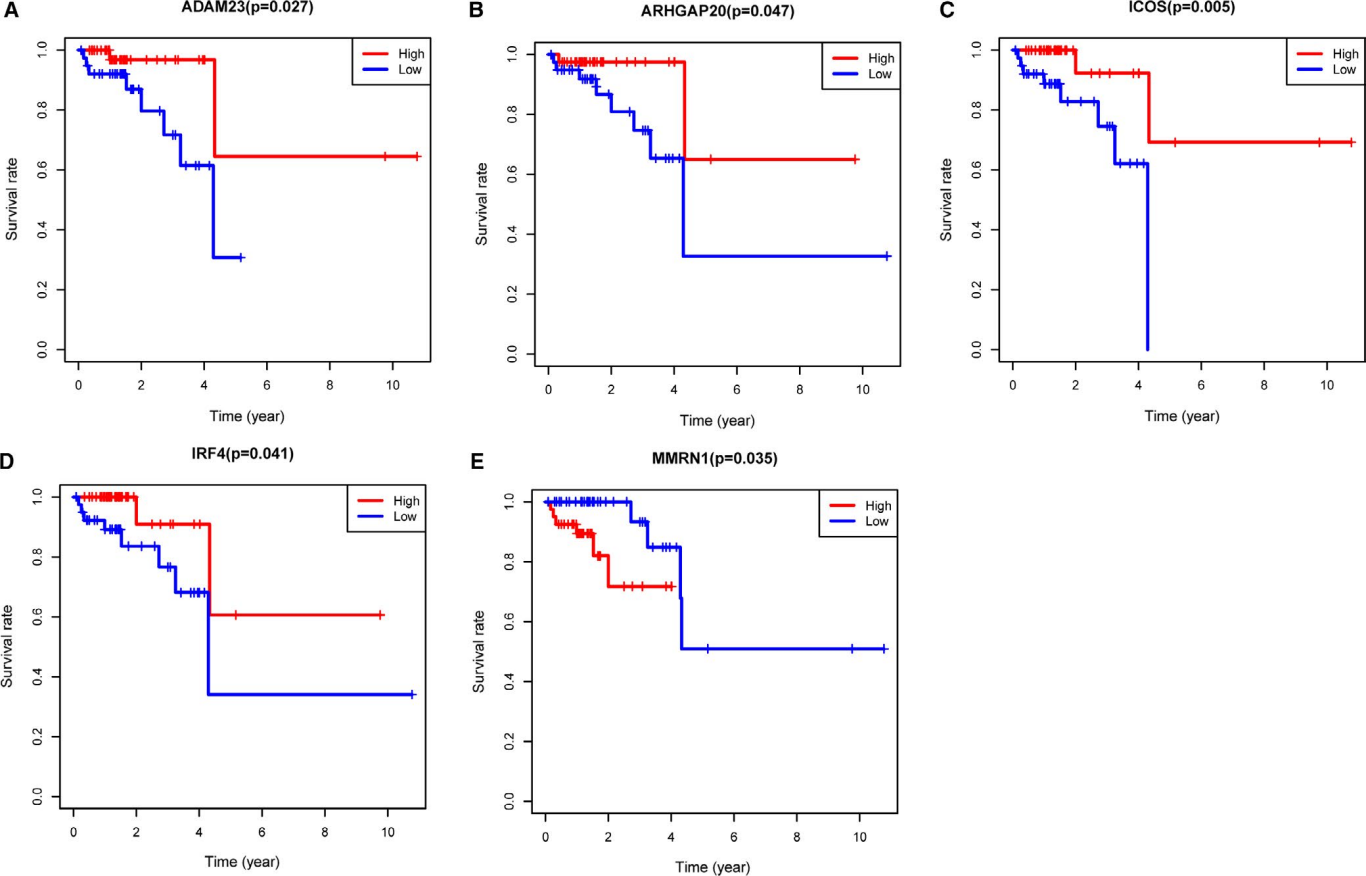
Five TCGA genes were verified to be related to OS

To explore the prognostic value of 540 DEGs, we performed the Kaplan‐Meier survival analysis. Among the 540 DEGs, a total of 60 DEGs (*P* < .05, Table S1) were significantly associated with OS (Figure 6), and all the genes were up‐regulated DEGs.

### 3.5 Module analysis from the PPI network

**FIGURE 7:**
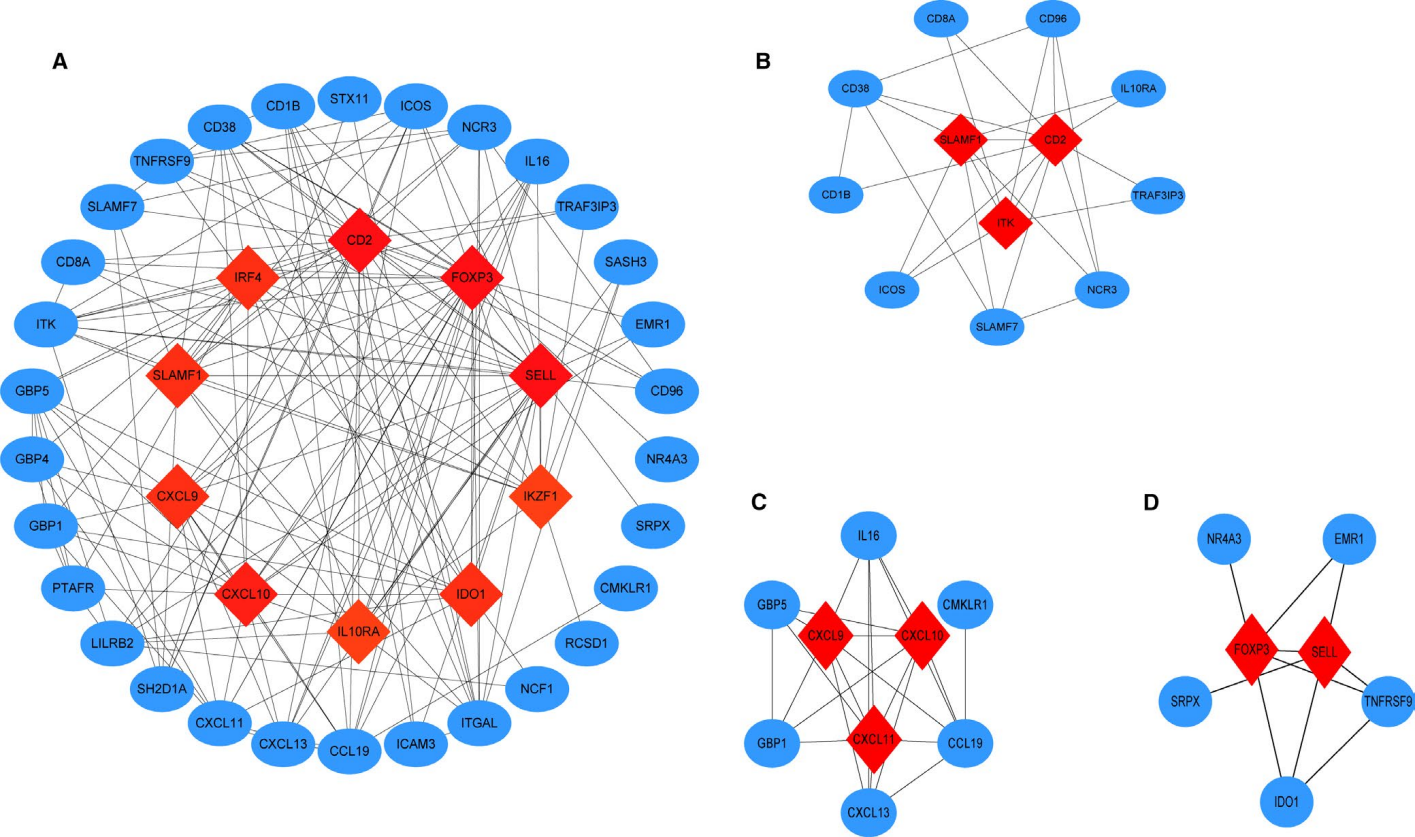
PPI network of prognostic DEGs and the top three significant modules. A, PPI network of the DEGs. B, PPI network of the 12 prognostic DEGs in Module 1. C, PPI network of the 9 prognostic DEGs in Module 2. D, PPI network of the 7 prognostic DEGs in Module 3

To further explore the interaction of the prognostic DEGs and the mechanisms underlying the rectal cancer development, we utilized the STRING online database and Cytoscape software to analyse these DEGs and construct a PPI network, which contains 40 nodes and 166 edges (Figure 7A). We then carried out clustering analysis and identified nine function modules. The top three significant modules were selected based on the degree of importance and the biological processes were analysed associated with the genes in the three modules.

Module 1 contains 12 nodes and 26 edges (Figure 7B). GO analysis revealed the 12 genes to be mainly enriched in the immune system process; KEGG pathway enrichment analyses demonstrated that the 12 genes are associated with haematopoietic cell lineage, T cell receptor signalling pathway and cell adhesion molecules. Module 2 contains 9 nodes and 23 edges (Figure 7C); GO and KEGG analysis indicates these 9 genes mainly enriched in chemokine signalling pathway and cytokine‐cytokine receptor interaction. Module 3 contains 7 nodes and 10 edges (Figure 7D), GO and KEGG analysis showed these 9 genes are associated with regulation of chronic inflammatory response and immune system process.

### 3.6 Validation of prognostic DEGs using the GEO database

**FIGURE 8:**
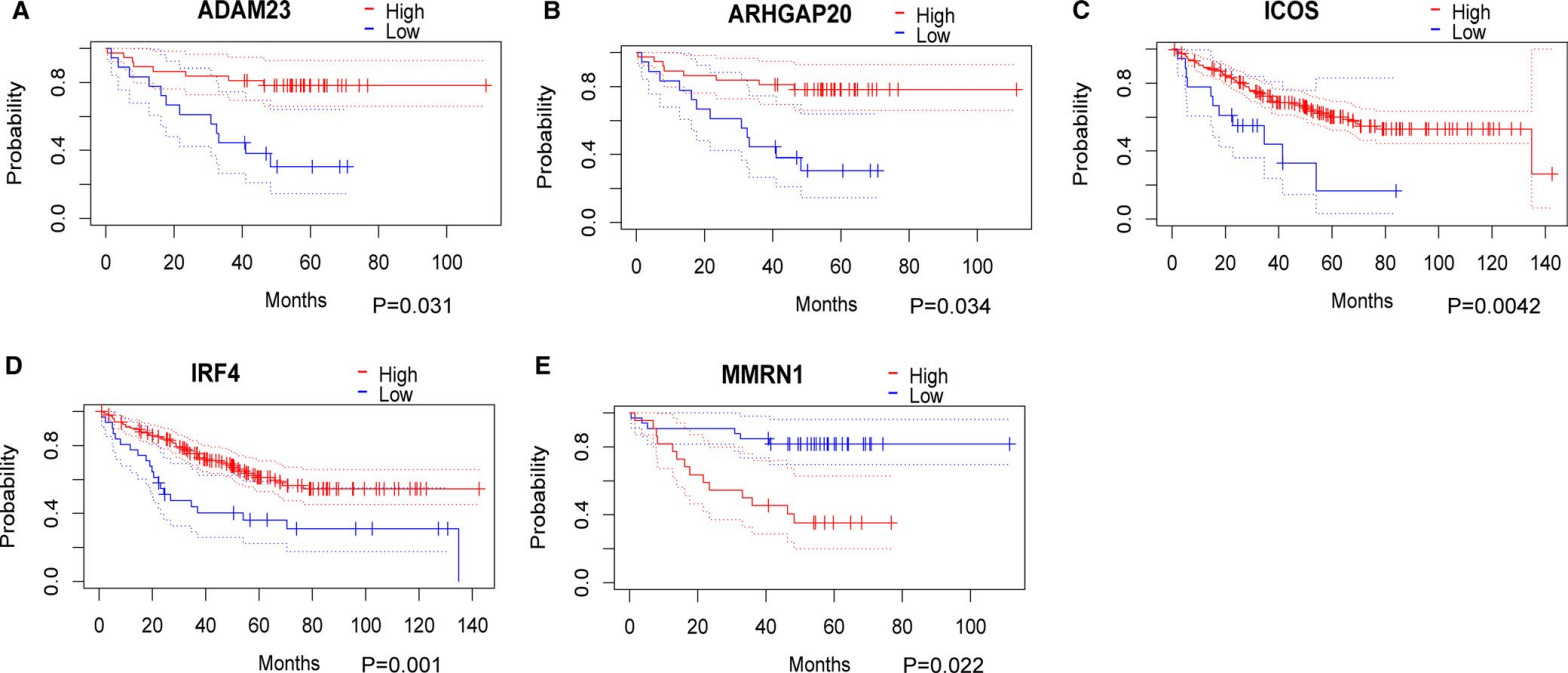
Validation of prognostic genes using data from the GEO database

To verify the DEGs from TCGA database, we downloaded the gene expression and prognostic information from the GSE17536 and GSE17537 data sets using PrognoScan online tool. A total of 19 prognostic genes were verified, which may have potential value for diagnosis and treatment of rectum cancer (Figure 8).

### 3.7 Verification of Prognostic DEGs using clinical tissue samples

**FIGURE 9:**
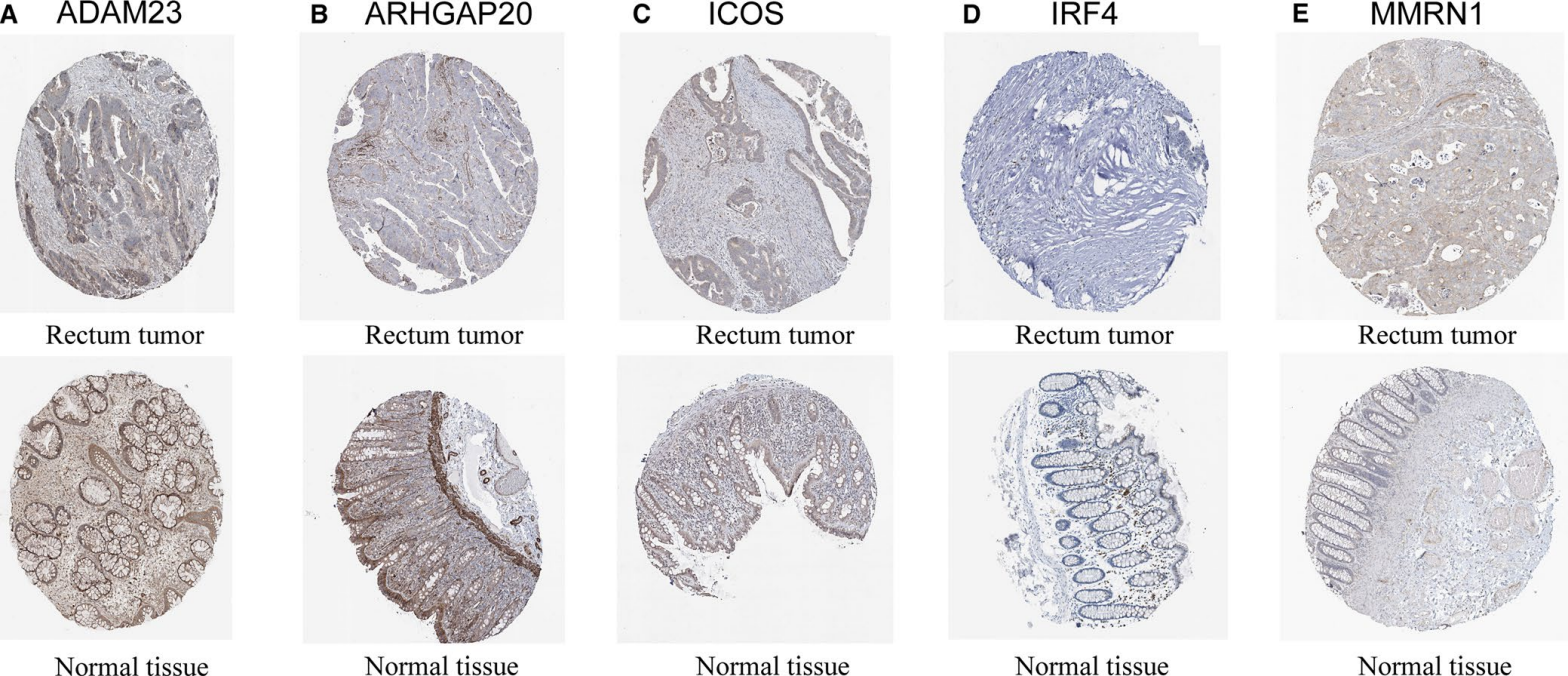
IHC analysis of genes with prognostic values

To verify the reliability of the DEGs with prognostic values, we detected the protein expression of 19 genes in normal tissues and tumour tissues by HPA. The results showed that 5 proteins (*ADAM23, ARHGAP20, ICOS, IRF4,* *MMRN1*) were significantly different in tumour tissues compared with normal tissues (Figure 9).

### 3.8 Expression of hub genes in cell types

**FIGURE 10:**
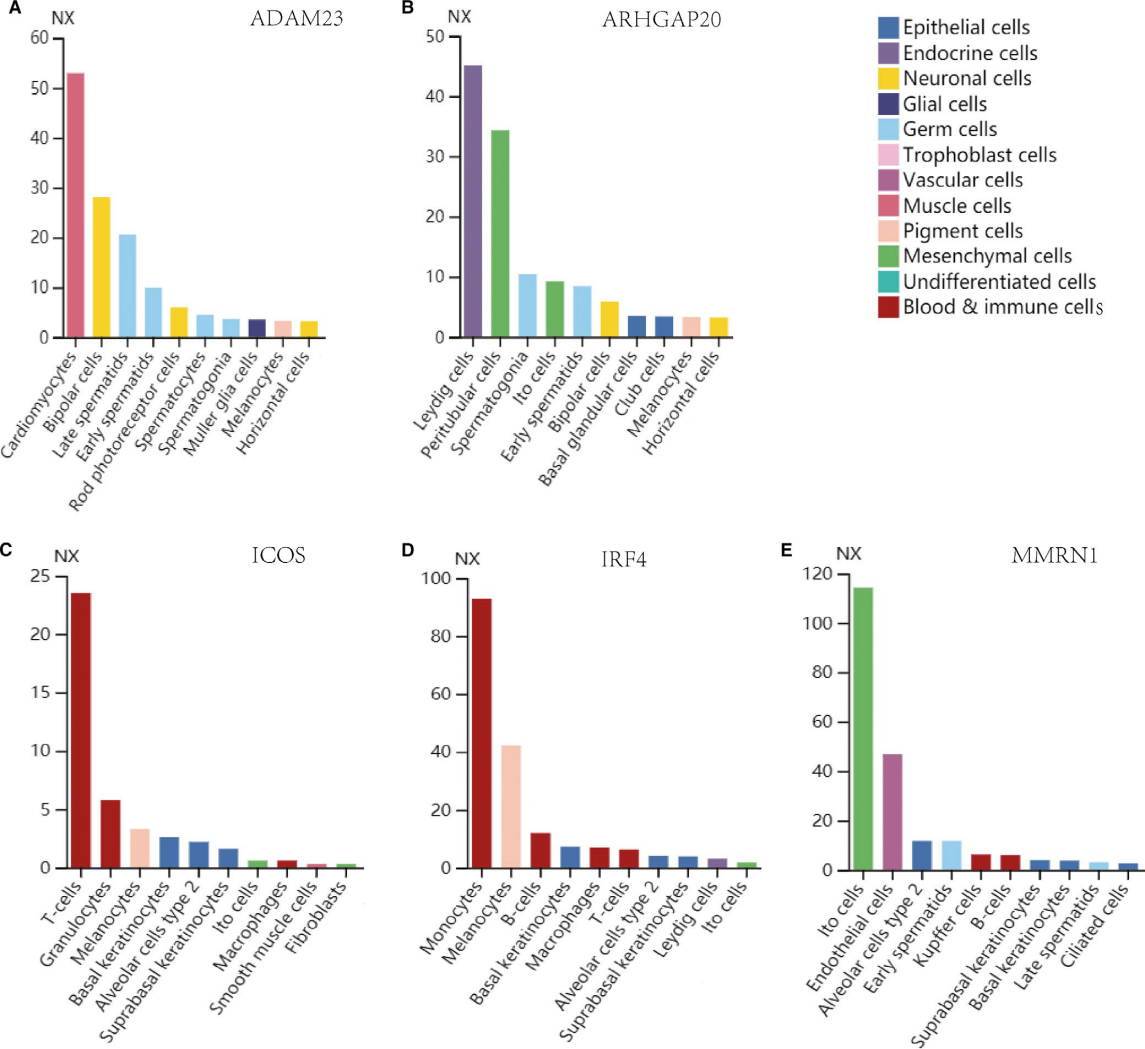
The expression of hub genes in cell types

The mRNA and protein levels of the 5 hub genes expression in cell types were evaluated, and the results showed that expression was predominantly found in blood and immune cells, mesenchymal cells, endocrine and germ cells cell types (Figure 10). Flow diagram of this study was listed in Figure S2.

## 4 DISCUSSION

In this study, we explored the level of stromal/immune cells and the relation between stromal/immune cells and overall survival of rectal cancer by the ESTIMATE algorithm and TCGA database. Moreover, we screened 540 tumour microenvironment‐related genes and explored prognostic biomarkers. Then we performed functional enrichment analysis and constructed the PPI network for exploring the potential mechanism of rectal cancer. The results showed that high‐immune score predicted a better prognosis in rectal cancer patients according to Kaplan‐Meier. In addition, a total of 60 DEGs were determined to be related with OS. Furthermore, GO and KEGG analyses revealed that DEGs are mainly enriched in the immune response, cytokine and chemokine activity. Finally, a total of 5 DEGs were verified GEO database and clinical tissue samples.

Tumour development is highly dependent on TME, which is consisted of extracellular matrix, stromal cells and immune cells, and any alterations of TME may influence the growth and progression of malignancies.^20^ However, current studies have not effectively analysed the components of TME of rectal cancer. ESTIMATE algorithm is a biology tool that based on expression signatures and Gene Set Enrichment Analysis to explore the stromal and immune cells in tumour samples.^14^ ESTIMATE has been successfully used in TME to determine immune/stromal cell consistence and their relations to clinical outcome in lung cancer,^21^, ^22^ breast cancer,^23^ ovarian cancer,^24^ renal cell carcinoma,^25^, ^26^ adrenocortical carcinoma,^27^ cutaneous melanoma,^28^ bladder cancer,^29^ endometrial cancer,^30^ pancreatic adenocarcinoma^31^ and osteosarcoma.^32^ It is the first study to evaluate the immune/stromal infiltration and their relations to prognosis and clinical outcomes in rectal cancer.

Firstly, we found patients with high‐immune score had a better OS, which may due to the positive correlation between the higher immune score and less tumour cell. In addition, the lower immune score was significantly related to the M1 stage and lymphatic invasion, indicating that lower immune scores indicated the advanced cancer stage and worse prognosis. These results were similar to previous studies, which have demonstrated that lower immune scores were significantly associated with poor overall survival in adrenocortical carcinoma,^27^ osteosarcoma^32^ and gastric cancer.^33^ However, some studies found that a higher immune score indicated a worse OS in clear cell renal cell cancer,^16^, ^17^, ^25^ and acute myeloid leukaemia.^34^ The underlying mechanism was not clear and required more explorations.

Then, we analysed the gene expression profiles and identified 540 DEGs and performed functional enrichment analysis. The GO functional analysis suggested that these DEGs were mainly involved in immune response, cytokine and chemokine activity. The KEGG pathway enrichment analysis showed that DEGs mainly clustered in cytokine‐cytokine receptor interaction and chemokine signalling pathway. These signalling immune cells and chemokines take an important role in the microenvironment and the progression of tumours. Chemokines were mediators of inflammation (inflammatory chemokines), which plays a key role in the progression of cancers,^35^ including the recruitment of different types of cell to the TME. Chemokines bind to the chemokine receptor subfamily, including 10 C‐C chemokine receptor (CCR) family members, 7 CXCR family members and CX3CR1.

Furthermore, we identified 60 prognostic genes by performing survival analysis of the 540 DEGs. The module analyses and function analyses showed these DEGs were mainly enriched in immune response, cytokine and chemokine activity. Then, we verified 5 hub genes as key prognostic biomarkers for rectal cancer. Among these genes, the higher expressions of *ADAM23*, *ARHGAP20*, *ICOS* and *IRF4* predicted better prognosis, while *MMRN1* predicted worse prognosis.

A disintegrin and metalloprotease 23 (*ADAM23*), a member of the ADAM family, is considered a possible tumour suppressor gene,^36^ and is frequently down‐regulated in various types of malignancies.^37^, ^38^ The silencing or decreased methylation of *ADAM23* gene often associated with advanced disease and metastasis in different types of tumours,^39^, ^40^ including colorectal cancer.^41^ ARHGAP family genes are cancer‐associated genes,^42^ and the genetic alterations of ARHGAP family genes lead to carcinogenesis.^43^ Previous studies showed the methylation of *ARHGAP20* is associated with prostate cancer,^44^ but the relation with gastrointestinal tumours is not clear. Inducible T‐cell co‐stimulator (*ICOS*) belongs to the B7‐CD28 immunoglobulin superfamily, which has dual role in different malignancies,^45^ and might participate in anti‐tumour T cell response as well as a pro‐tumour response.^46^ A significant down‐regulation of *ICOS* can be seen in colon cancer patients,^47^ especially in patients with either lymphatic or distant metastasis. Conclusively, expression of *ICOS* is associated with improved survival in colorectal cancer.^48^ Interferon regulatory factor 4 (*IRF4*), a member of the interferon regulatory factor family of transcription factors, is expressed in cells of the immune system. *IRF4* have critical roles in the immunosuppressive tumour microenvironment,^49^ and the deficiency of *IRF4* accelerates tumour growth and reduces survival in pancreatic cancer.^50^ Studies demonstrated that *IRF4* was associated with rectal cancer.^51^ Multimerin1 (*MMRN1*) is a di‐sulphide linked homo‐polymeric glycoprotein from EMILIN family. Altered expression of *MMRN1* has been reported in hepatocellular carcinoma, cervical cancer and ovarian cancer.^52^, ^53^ *MMRN1* also played an important risk factor in gastric cancer microenvironment.^54^ But its role in rectal cancer is not clear.

Our findings have certain clinical implications. Firstly, the expression level of DEGs in rectal tumour tissue may contribute to the prognosis prediction and the evaluation of survival. For example, patients with a high expression of protective genes may have good prognosis and longer survival. Secondly, the DEGs could help guide personalized treatment. For patients with a high expression of risk genes, it is worthwhile to perform detailed examinations and aggressive treatments to prevent tumour metastasis and lymphatic invasion. Thirdly, these genes have functional relevance in rectal cancer, which could contribute to the search for biomarkers and therapeutic targets. Besides, these genes are promising biomarkers in rectal cancer because of highly stability and non‐invasive biopsy.

There are some limitations in this study. Firstly, the selection bias could not be excluded because all data were gathered from TCGA and GEO databases. Secondly, there was no experimental research to examine the functions of DEGs. Thus, further validation is needed to testify the discovery of this research.

In summary, we found that stromal and immune scores were highly associated with the clinical outcome of rectal cancer by ESTIMATE algorithm and TCGA database. In addition, we identified 5 microenvironment‐related genes which could be useful for outlining the prognosis of rectal cancer. The results could contribute to the search for rectal cancer biomarkers and therapeutic targets.

## CONFLICTS OF INTEREST

There are no conflicts of interest.

## AUTHOR CONTRIBUTIONS

**Chao Li:** Data curation (supporting); Formal analysis (supporting); Methodology (supporting); Software (supporting); Writing‐original draft (supporting); Writing‐review & editing (supporting). **Tao Liu:** Formal analysis (supporting); Writing‐review & editing (supporting). **Yi Liu:** Software (supporting); Validation (supporting). **Jiantao Zhang:** Conceptualization (supporting); Project administration (lead). **Didi Zuo:** Data curation (lead); Formal analysis (lead); Methodology (lead); Software (lead); Writing‐original draft (lead); Writing‐review & editing (lead).

## Supporting information

Figure S1

Figure S2

Table S1

## Data Availability

The data that support the findings of this study are available from the corresponding author upon reasonable request.
