# Supplementary figures and images for: Prognostic value of tumour microenvironment‐related genes by TCGA database in rectal cancer
Source: J Cell Mol Med. 2021 May 5;25(12):5811–22. doi: 10.1111/jcmm.16547 (PMC8184694; doi:10.1111/jcmm.16547)

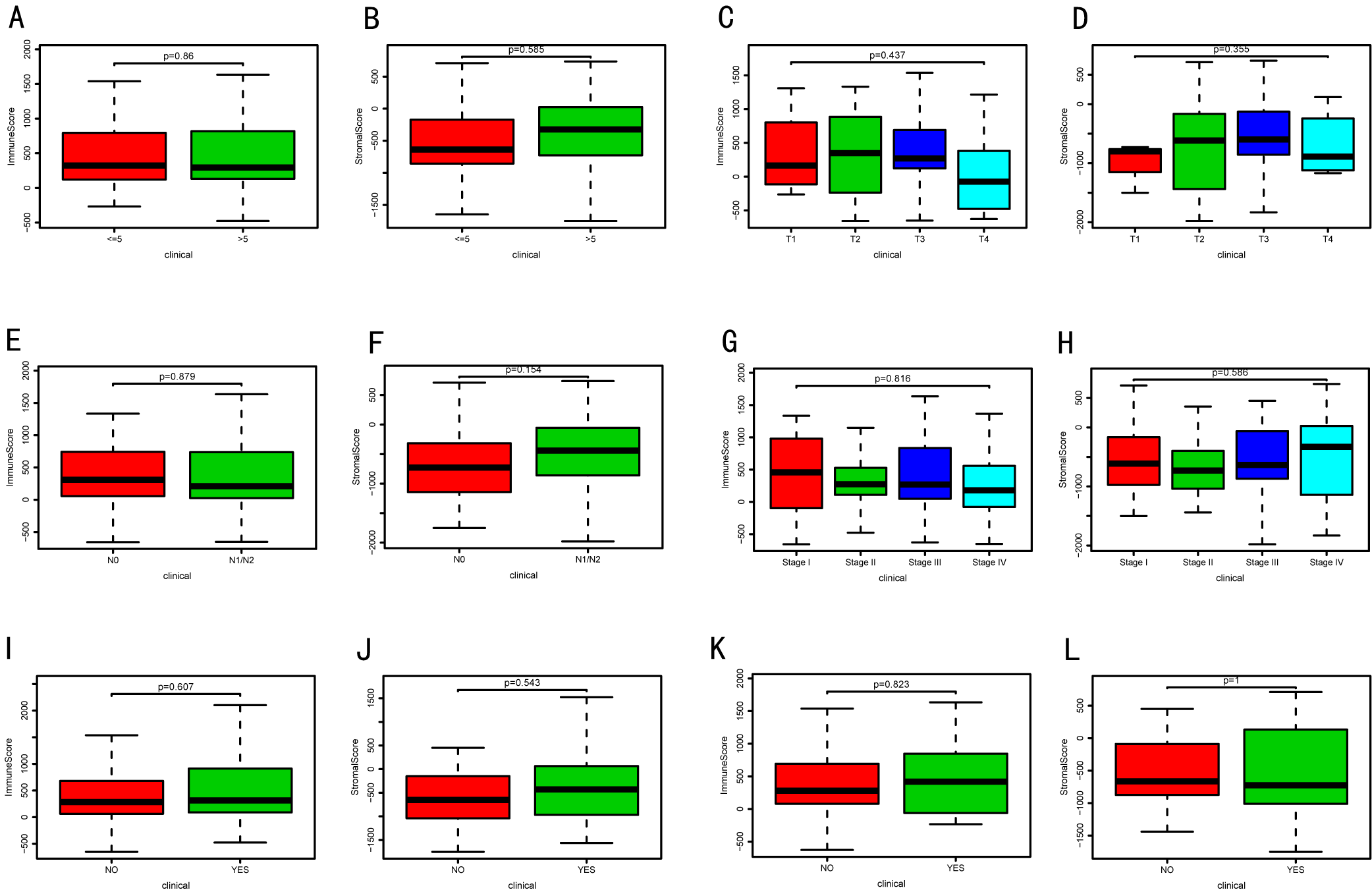

Supplement: Supplementary file 1 — Figure S1 [file JCMM-25-5811-s002.tif]

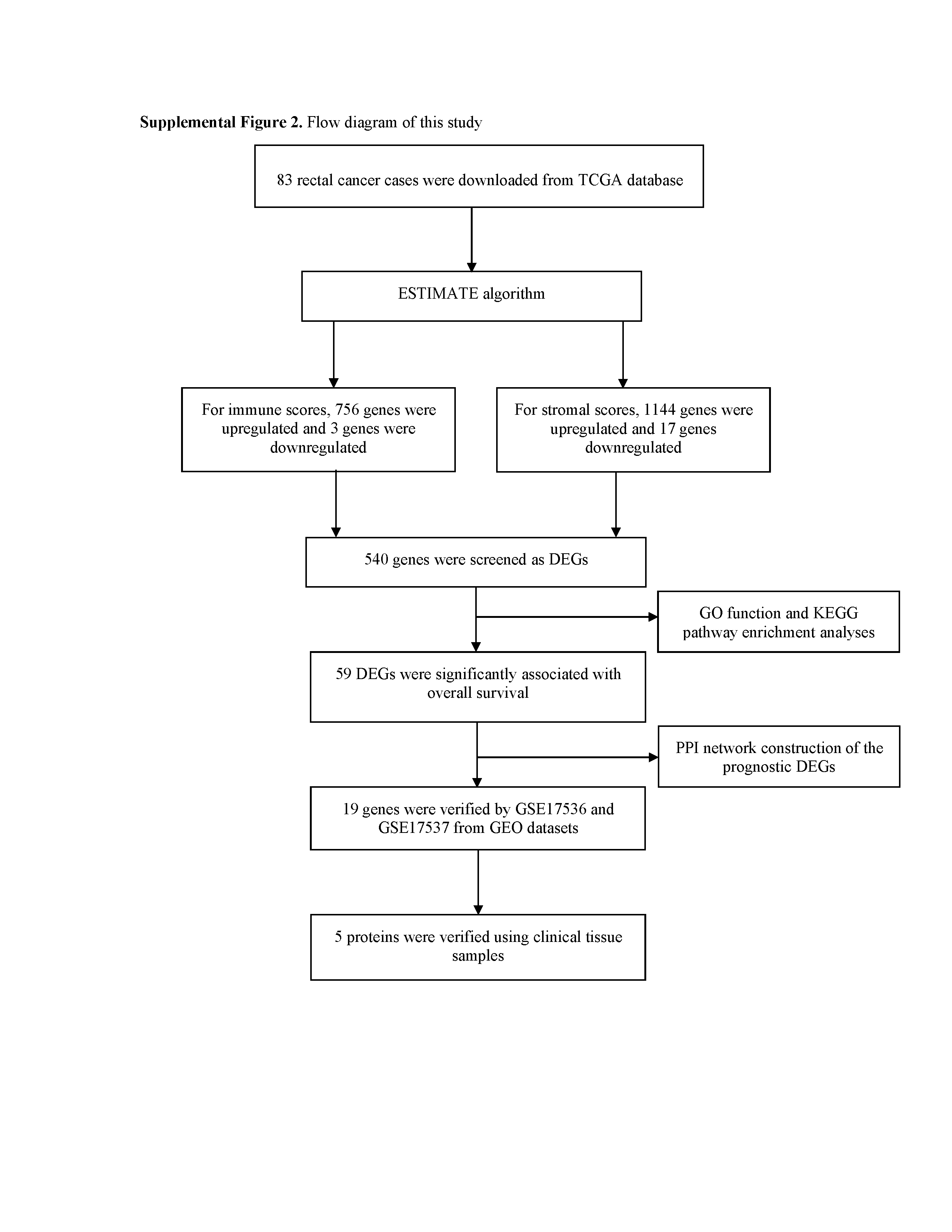

Supplement: Supplementary file 2 — Figure S2 [file JCMM-25-5811-s001.tif]
