# Supplementary material for: Prognostic value of tumour microenvironment‐related genes by TCGA database in rectal cancer
Source: J Cell Mol Med. 2021 May 5;25(12):5811–22. doi: 10.1111/jcmm.16547 (PMC8184694; doi:10.1111/jcmm.16547)
Supplement: Supplementary file 3 — Table S1 [file JCMM-25-5811-s003.docx]

Supplemental Table 1. 60 DEGs were significantly associated with overall survival (*P*＜0.05).

| **Gene** | Logrank P | **HR** | **HR.95L** | **HR.95H** | ***P*** | **Gene** | Logrank P | **HR** | **HR.95L** | **HR.95H** | ***P*** |
| --- | --- | --- | --- | --- | --- | --- | --- | --- | --- | --- | --- |
| **ZBED2** | 0.014 | 0.985 | 0.817 | 1.186 | 0.871 | **CD274** | 0.042 | 0.048 | 0.002 | 0.916 | 0.044 |
| **CYR61** | 0.027 | 0.981 | 0.945 | 1.017 | 0.292 | **PTAFR** | 0.022 | 0.327 | 0.115 | 0.927 | 0.036 |
| **GZMK** | 0.044 | 0.235 | 0.033 | 1.666 | 0.147 | **PLA2G2D** | 0.007 | 0.093 | 0.006 | 1.410 | 0.087 |
| **MMRN1** | 0.035 | 0.989 | 0.405 | 2.415 | 0.981 | **CD4** | 0.045 | 0.785 | 0.587 | 1.050 | 0.103 |
| **MIXL1** | 0.013 | 0.000 | 0.000 | 10.079 | 0.101 | **LAX1** | 0.023 | 0.001 | 0.000 | 0.289 | 0.019 |
| **FOXP3** | 0.003 | 0.542 | 0.246 | 1.192 | 0.128 | **PYHIN1** | 0.041 | 0.001 | 0.000 | 1.175 | 0.055 |
| **CMKLR1** | 0.026 | 0.154 | 0.024 | 0.977 | 0.047 | **GBP5** | 0.004 | 0.039 | 0.002 | 0.944 | 0.046 |
| **ADAM23** | 0.027 | 0.020 | 0.000 | 3.498 | 0.138 | **CXCL10** | 0.016 | 0.918 | 0.833 | 1.013 | 0.087 |
| **ADGRE1** | 0.042 | 0.000 | 0.000 | 2.562 | 0.076 | **LRFN1** | 0.032 | 0.849 | 0.439 | 1.642 | 0.626 |
| **MZB1** | 0.041 | 0.644 | 0.374 | 1.110 | 0.113 | **CD96** | 0.012 | 0.017 | 0.000 | 0.749 | 0.035 |
| **IL16** | 0.032 | 0.111 | 0.006 | 2.236 | 0.151 | **ICOS** | 0.005 | 0.004 | 0.000 | 0.299 | 0.012 |
| **TRAT1** | 0.026 | 0.000 | 0.000 | 0.926 | 0.048 | **IL10RA** | 0.014 | 0.366 | 0.126 | 1.065 | 0.065 |
| **SASH3** | 0.033 | 0.632 | 0.390 | 1.022 | 0.061 | **SLAMF1** | 0.010 | 0.006 | 0.000 | 0.419 | 0.018 |
| **TNFRSF9** | 0.004 | 0.001 | 0.000 | 0.239 | 0.014 | **SIRPB1** | 0.038 | 0.001 | 0.000 | 2.775 | 0.089 |
| **TRAF3IP3** | 0.031 | 0.011 | 0.000 | 0.929 | 0.046 | **SELL** | 0.013 | 0.401 | 0.144 | 1.111 | 0.079 |
| **LILRB2** | 0.041 | 0.405 | 0.106 | 1.546 | 0.186 | **LY9** | 0.049 | 0.005 | 0.000 | 30.800 | 0.237 |
| **CCL22** | 0.028 | 0.390 | 0.146 | 1.041 | 0.060 | **IKZF1** | 0.028 | 0.025 | 0.001 | 0.602 | 0.023 |
| **CXCL13** | 0.004 | 0.396 | 0.167 | 0.939 | 0.035 | **IRF4** | 0.041 | 0.006 | 0.000 | 0.412 | 0.017 |
| **CXCL9** | 0.007 | 0.793 | 0.634 | 0.993 | 0.043 | **ZNF683** | 0.025 | 0.136 | 0.005 | 4.050 | 0.249 |
| **ITGAL** | 0.006 | 0.320 | 0.103 | 1.000 | 0.050 | **CD2** | 0.019 | 0.679 | 0.459 | 1.006 | 0.054 |
| **NCR3** | 0.020 | 0.001 | 0.000 | 0.448 | 0.027 | **SH2D1A** | 0.008 | 0.001 | 0.000 | 0.274 | 0.016 |
| **ICAM3** | 0.033 | 0.004 | 0.000 | 0.283 | 0.011 | **ARHGAP20** | 0.047 | 0.000 | 0.000 | 32.703 | 0.144 |
| **RHOH** | 0.034 | 0.032 | 0.001 | 1.085 | 0.056 | **CASS4** | 0.047 | 0.000 | 0.000 | 1.112 | 0.053 |
| **ITK** | 0.043 | 0.002 | 0.000 | 0.638 | 0.035 | **RAB39B** | 0.027 | 0.000 | 0.000 | 2.491 | 0.065 |
| **APBB1IP** | 0.042 | 0.147 | 0.023 | 0.923 | 0.041 | **SLAMF7** | 0.023 | 0.306 | 0.100 | 0.931 | 0.037 |
| **CD38** | 0.025 | 0.900 | 0.503 | 1.610 | 0.722 | **MEFV** | 0.013 | 0.001 | 0.000 | 10.157 | 0.139 |
| **FCRLA** | 0.037 | 0.023 | 0.000 | 3.311 | 0.137 | **STX11** | 0.041 | 0.394 | 0.092 | 1.683 | 0.209 |
| **GBP1** | 0.003 | 0.707 | 0.520 | 0.961 | 0.027 | **CD8A** | 0.024 | 0.290 | 0.088 | 0.949 | 0.041 |
| **CD1B** | 0.021 | 0.000 | 0.000 | 1.049 | 0.051 | **KLHDC7B** | 0.004 | 0.001 | 0.000 | 0.397 | 0.024 |
| **GBP4** | 0.012 | 0.535 | 0.293 | 0.978 | 0.042 | **IDO1** | 0.007 | 0.470 | 0.218 | 1.013 | 0.054 |
